# Effects of Ankle–Foot Orthoses on Functional Recovery after Stroke: A Propensity Score Analysis Based on Japan Rehabilitation Database

**DOI:** 10.1371/journal.pone.0122688

**Published:** 2015-04-02

**Authors:** Ryo Momosaki, Masahiro Abo, Shu Watanabe, Wataru Kakuda, Naoki Yamada, Shoji Kinoshita

**Affiliations:** Department of Rehabilitation Medicine, The Jikei University School of Medicine, Tokyo, Japan

## Abstract

**Objectives:**

The purpose of the present study was to investigate potential effects of ankle–foot orthoses (AFOs) on the functional recovery of post-acute stroke patients following rehabilitation.

**Subjects and Methods:**

This study is a retrospective cohort study. Participants were in-hospital stroke patients registered in the Japan Rehabilitation Database between 2005 and 2012. A total of 1862 patients were eligible after applying exclusion criteria. Propensity score analysis was applied to adjust for potential bias and to create two comparable groups. An additional subset analysis focused on Functional Independence Measure (FIM) scores on admission.

**Results:**

In this sample, 30.7% of 1863 eligible patients were prescribed AFOs. Propensity score matched analysis showed that patients with AFOs had significantly higher scores than those without them for discharge FIM (mean: 91.3 vs 85.8; *p*=0.02), FIM gain (mean: 28.9 vs 23.5; *p*<0.001), and FIM efficiency (mean: 0.27 vs 0.22; *p*<0.001). Inverse probability weighting analysis showed similar results. In the subset analysis, patients with AFOs had significantly higher discharge FIM compared with those without them in the low admission FIM subgroup only. In addition, patients with AFOs performed independent exercise more than those without them (*p*<0.001).

**Conclusions:**

These data suggest that stroke survivors may have better functional recovery if they are prescribed an AFO than if they are not prescribed an AFO. The use of AFOs is considered to be a feasible option to improve functional recovery of stroke rehabilitation patients.

## Introduction

Previous recommendations identify the use of ankle–foot orthoses (AFOs) as a feasible option in the rehabilitation of patients with hemiplegia after stroke. A National Health Service Quality Improvement Scotland scoping exercise identified the use of AFOs following stroke as a clinical improvement priority, leading to the development of a best practice statement on AFO use after stroke [1]. Nevertheless, some rehabilitation practitioners avoid using AFOs due to personal preferences.

Biomechanical gait analyses have shown AFOs to improve ankle and knee kinematics in stroke patients [2]. A previous meta-analysis showed that AFOs improve walking ability, gait speed, and balance after stroke [3]. In addition, another report suggested that AFOs potentially reduce the risk of falls in stroke patients with hemiplegia [4].

However, many clinical studies only examined the immediate effects of AFOs [3], and there is no relevant literature on the effectiveness of employing conventional AFOs in the post-acute stroke recovery stage on rehabilitation outcomes. It appears difficult in practice to conduct a randomized controlled trial to examine their effectiveness because of their widespread use; instead, a large-scale observational study using propensity score analysis methods is a feasible alternative to a randomized controlled trial. We hypothesized that AFOs—which make it easy to do independent exercise and improve the motivation to exercise and the quality of exercise—affect the overall ability to engage in activities of daily living. This retrospective observational study aimed to determine the effects of conventional AFO use on the functional recovery of post-acute stroke patients using propensity score analysis methods and the Japan Rehabilitation Database.

## Materials and Methods

### Japan Rehabilitation Database

The Japan Rehabilitation Database has been developed with financial support from the Ministry of Health, Labour, and Welfare of Japan [5, 6]. Detailed clinical data was collected for rehabilitation inpatients discharged from participating hospitals (not all of which have a dedicated rehabilitation unit) from 2005. The Japan Rehabilitation Database consists only of voluntary samples, not random samples. The database includes unique identifiers for the following stroke patient data: age and sex; Functional Independence Measure score (FIM: scores range from 18 [totally dependent] to 126 [totally independent]) [7]; length of stay; days from onset; type of stroke; amount of exercise; 7-grade Brunnstrom recovery stage (BRS: level 1 [completely flaccid paralysis] to level 7 [without paralysis]) [8]; 5-grade modified Rankin Scale (mRS: level 1 [no significant disability] to level 5 [severe disability]) [9], and; prescription of AFOs. Rehabilitation staff collected baseline data at admission. Variables not collected at admission were collected at discharge. These data were submitted to the Japan Association of Rehabilitation Database. The Association extracted the data and sent it to us.

By 2013, 78 hospitals had contributed structured data for 29,339 patients to the database. All personal data were coded deleting any information related to personal identification. Patients did not provide informed consent for inclusion of their data in the database and subsequent use of the data in research investigations. Informed consent was waived because of the anonymous nature of the data. The Institutional Review Board of The Japanese Association of Rehabilitation Medicine approved this study. Ethical approval had previously been granted by the Japanese Association of Rehabilitation Medicine; the reference is available on website of Japan Association of Rehabilitation Database [10].

We confirmed that some access restrictions apply to the data underlying the findings. Because the dataset of this study is owned by the Japan Association of Rehabilitation Database, we are unable to make the entire dataset publicly available. However, the Japan Association of Rehabilitation Database will receive appropriate requests for all relevant data.

### Subjects

This study included patients admitted to the convalescent rehabilitation wards [11] of 33 participating rehabilitation hospitals with a diagnosis of stroke between January 2005 and October 2012, sourced from the Japan Rehabilitation Database. Other inclusion criteria were that the patients: (1) were aged between 20 and 90 years old; (2) were within 90 days of stroke onset at admission; (3) stayed between 30 and 180 days; (4) had been diagnosed with either hemorrhagic or ischemic stroke; (5) had a premorbid mRS of 1–3 (i.e., without severe disability before onset); (6) had a BRS of the lower extremities on admission of 1 or 5 (i.e., mild to severe paralysis); and (7) had complete data. We focused on adults, excluding very elderly people, who had a good recovery period. We excluded patients for whom AFO indication was slight, i.e., those who already had a severe disability before onset, and had only slight or no paralysis in the lower extremities. With respect to the length-of-stay inclusion criterion, in the Japanese medical insurance system, the maximum coverage with respect to the length of stay for stroke patients with rehabilitation is 180 days. It takes 2 weeks from the date of order for an AFO to be supplied. We considered that more than 2 weeks of exercise with AFO were required to estimate functional recovery effect of AFO (i.e., there is not an immediate effect), hence to be included in the study patients must have stayed for at least 30 days. Convalescent rehabilitation wards are the main system providing inpatient rehabilitation facilities covered by Japan’s medical insurance system. Patients who still need assistance in activities of daily living after acute treatment are transferred to these rehabilitation wards [11]. The maximum length of stay and amount of exercise with therapists covered by insurance are limited, with the length of stay and amount of exercise with therapists per day determined by a rehabilitation physician on admission systematically according to the degree of disability.

### Rehabilitation program

The typical gym exercise program was composed of 40 minutes of physical therapy and 40 minutes of occupational therapy per day, 5–7 days per week [12]. Physical rehabilitation focused on gait and exercise related to activities of daily living. The program included passive range of motion exercises for the affected side and muscle strengthening exercises for the unaffected side. Speech therapy was also performed if necessary. In addition, patients were encouraged to stay out of bed during the day. Typical independent exercise, such as standing and walking under the supervision of nurses, was performed for 20–30 minutes per day, 5–7 days per week (without therapists present). Attending physicians, taking into consideration the patients’ character, motivation, and level of disability, approved their independent exercise programs. Our data indicate only whether patients did independent exercise or not during hospitalization, and not the quality or quantity of such exercise. In this study, no detailed data were available regarding the rehabilitation program that was provided for the patients. The Japan Rehabilitation Database does not have this information. The program for the patients might have varied among the studied patients because the criteria for this study did not refer to the content of the rehabilitation program.

### Ankle–foot orthosis

Subjects were divided into two groups: the AFO group and the No-AFO group. The AFO group was defined as patients who were prescribed an AFO during hospitalization for in-hospital exercise. The No-AFO group was not prescribed AFOs during hospitalization. A rehabilitation physician prescribes AFOs for patients in Japan. AFOs are covered under health insurance. However, patients must cover some of the expenses under their consent.

### Outcome measurements

The primary clinical outcome was discharge FIM [7] (FIM on discharge). FIM is a basic indicator of disability severity. The FIM consists of 18 items, each of which is assessed against a seven-point ordinal scale; the higher the score for an item, the more independently the patient is able to perform the tasks assessed by that item. The secondary clinical outcomes were FIM gain (discharge FIM—admission FIM), FIM efficacy (FIM gain / length of stay) [13], and amount of independent exercise.

### Data analysis

This study performed one-to-one matching between the AFO and No-AFO groups based on estimated propensity scores for each patient [14]. In this study, the biggest problem was selection bias. AFO prescription (indication) is greatly influenced by subject characteristics. As a result, the characteristics of the AFO group differ systematically from the No-AFO group. We must therefore account for these systematic differences in characteristics between the two groups when estimating the effects of AFOs on the outcomes. Historically, researchers have relied on the use of regression adjustment to account for differences in measured characteristics between two groups. However, it is generally difficult to analyze causal effects of treatment in an observational study due to treatment selection bias. Therefore, we decided to use propensity score analysis to remove the selection bias and ensure comparability. The propensity score is the probability of treatment assignment conditional on observed characteristics. In particular, the propensity score is a balancing score: after applying it, the distribution of observed covariates will be similar between treated and untreated subjects. The propensity score matching method attempts to simulate a randomized experimental situation, in which both groups are comparable in observed prognostic factors.

We also performed a post hoc power analysis to ascertain the statistical validity of our sample size. Values are expressed as mean ± standard deviation (SD). The threshold for significance was set at *p*<0.05. Statistical analyses were conducted using IBM SPSS version 22.0 (IBM Corp., Armonk, NY, US).

### Quasi-experimental design using propensity score methods

A quasi-experimental design is created when the probability (propensity score) that a patient would have been treated is used to adjust the estimate of the treatment effect [15]. It takes four steps to construct a quasi-experimental design using propensity score matching: (1) propensity score estimation; (2) matching algorithm selection; (3) balance checking; and (4) effect estimation.

In observational studies, the true propensity score is not known. However, it can be estimated using the data. In practice, the propensity score is most often estimated using a logistic regression model, in which treatment status is regressed against observed characteristics. To estimate the propensity score for the prescription of AFOs, we used a logistic regression model incorporating the following demographic factors: age, admission FIM, length of stay, days from stroke onset, type of stroke (hemorrhage or infarction), total amount of gym exercise per day, BRS of lower extremities on admission, and premorbid mRS. The variables measured included confounding variable and those related to exposure and outcome [16]. Baseline data (days from onset, type of stroke, BRS of lower extremities on admission, premorbid mRS, sex, and age) were collected at admission. Prescription of AFO, length of stay, total amount of gym exercise per day and discharge FIM were collected at discharge. It was assumed that AFO prescription greatly depended on the preferences of individual therapists and hospitals. These preferences caused a within-center correlation with the response variables. Such correlation or clustering occurs when outcomes within centers tend to be more similar to each other than to outcomes in other centers. We treated with the within-hospital correction using generalized estimation equations that are commonly used to analyze clustered data while correcting for confounding by cluster [17]. We used the c-statistic [18] to assess how well the propensity score differentiated those who received an AFO prescription from those who did not.

To select the matching algorithm, the nearest neighbor one-to-one method was used with the closest estimated propensity score within a caliper (≤0.25 of the pooled standard deviation of estimated logits). This algorithm is one of the most popular methods for propensity score matching [19]. Propensity score matching involves forming matched sets of treated and untreated subjects that share a similar propensity score.

Descriptive statistics are presented for all patients and the propensity-matched groups to check covariate balance and estimate effect. T-test and chi-square tests were employed to perform comparisons of the AFO and No-AFO groups. After patient matching, a subset analysis focused on admission FIM, with patients divided by the median FIM into the high FIM group (63 or over) or low FIM group (under 63). The purpose of this subset analysis was to assess whether differences in age and activities of daily living on admission influenced the effectiveness of the AFOs.

The propensity score matching method has a generalizability problem, due to the loss of unmatched subjects. We therefore checked the robustness of our results by applying inverse probability weighting to the propensity score, using all eligible sample data [20]. Inverse probability weighting is a propensity score method that uses weights based on the propensity score to create a synthetic sample in which the distribution of measured covariates is independent of treatment assignment.

## Results

**Fig 1:**
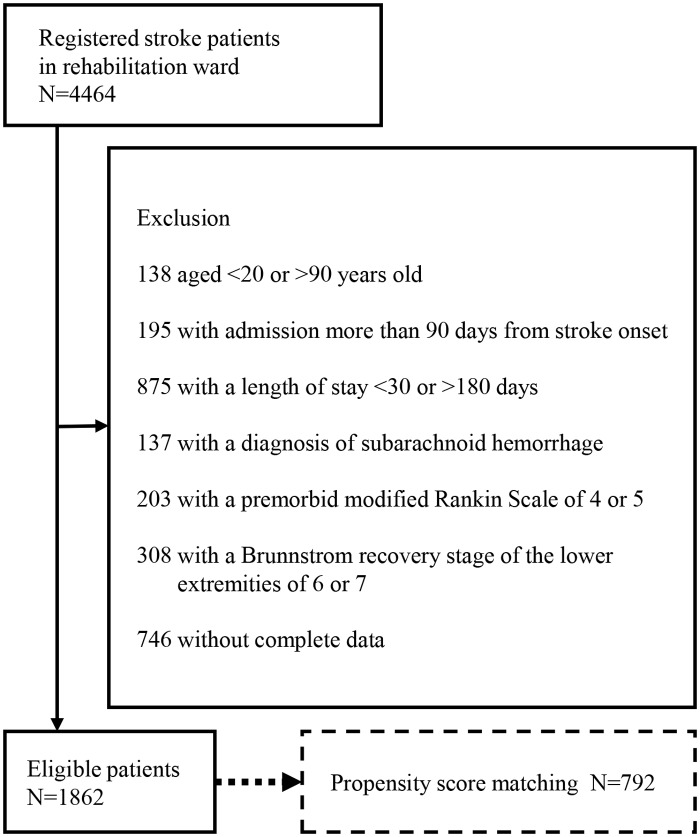
Patient selection from data.

A total of 4464 stroke patients were identified during the study period. Of the total cohort, the study excluded 138 patients aged under 20 or over 90 years, 195 who were more than 90 days past stroke onset, 875 who has resided in a rehabilitation hospital for less than 30 or more than 180 days, 137 diagnosed with subarachnoid hemorrhage, 203 whose premorbid mRS values were 4 or 5, 308 with a BRS of the lower extremities of 6 or 7, and 746 patients who had incomplete data (Fig. 1). As a result, there were 1862 patients left eligible for analysis. Table 1 shows the patient characteristics. The AFO prescription rate was 30.7%. The average age was over 65 years, and more than 90% of patients did not have premorbid disability (mRS≥2). Average FIM gain was approximately 25.

**Table 1:** Clinical characteristics of patients.

	Stroke patients (n = 1862)
Prescription of ankle–foot orthoses, n (%)	572 (30.7)
Mean age ± SD (years)	67.9±13.5
Sex (male), n (%)	113 (60.7)
Mean length of stay ± SD (days)	106.2±40.9
Mean days from onset ± SD (days)	33.0±17.3
Ischemic stroke, n (%)	895 (48.1)
Premorbid disability (mRS≥2), n (%)	143 (7.7)
Mean daily amount of gym exercise ± SD (minutes/day)	106.5±37.4
Mean admission FIM ± SD	66.5±29.2
Mean discharge FIM ± SD	91.2±30.4

FIM: Functional Independence Measure; mRS: modified Rankin Scale.

Table 2 shows patient demographics of the AFO and No-AFO groups for all patients (n = 1862) and the propensity score-matched patients (n = 792). Within the total population, the study identified 572 patients with AFOs and 1290 patients without AFOs. Using one-to-one propensity score matching, 396 pairs from the AFO and No-AFO groups were selected. The C-statistic for goodness of fit was 0.78 in the propensity score model. In the unmatched groups, AFO patients were younger, had longer hospital stays, lower admission FIM, and exercised more. After propensity score matching, patient distributions were closely balanced between the AFO and No-AFO groups.

**Table 2:** Patients characteristics by unmatched and propensity score-matched groups.

	Unmatched Groups			Matched Groups		
	AFO (n = 572)	No- AFO (n = 1290)	p value	AFO (n = 396)	No- AFO (n = 396)	p value
Mean age ± SD (years)	67.8±13.4	69.2±13.3	<.01	66.4±14.0	65.7±31.4	.45
Sex (male), n (%)	344 (60.1)	786 (60.9)	.75	226 (57.1)	226 (57.1)	1.0
Mean length of stay ± SD (days)	124.1±36.8	98.2±40.3	<.01	116.8±41.0	115.5±37.3	.64
Mean days from onset ± SD (days)	33.2±16.6	32.9±17.7	.76	33.0±17.4	32.5±16.6	.84
Ischemic stroke, n (%)	252 (44.1)	643 (49.8)	<.01	172 (43.4)	179 (45.2)	.97
Premorbid disability (mRS≥2), n (%)	31 (5.4)	112 (8.7)	.15	18 (4.5)	21 (5.3)	.54
Mean daily amount of gym exercise ± SD (minutes/day)	113.5±34.4	103.4±38.2	<.01	113.5±34.0	113.3±34.2	.92
Mean admission cognitive FIM ± SD	21.7±9.0	22.3±9.7	.16	21.8±8.9	22.8±9.9	.15
Mean admission FIM ± SD	59.7±24.8	69.5±30.4	<.01	62.0±32.0	62.4±25.8	.86

AFO: Ankle–foot orthosis; FIM: Functional Independence Measure; mRS: modified Rankin Scale.

Table 3 shows discharge FIM for the AFO and No-AFO groups. In the propensity-matched groups, the AFO group had significantly higher scores than the No-AFO group in discharge FIM (91.3±26.9 vs 85.8±35.2; *p* = 0.02), FIM gain (28.9±17.4 vs 23.5±21.0; *p*<0.001), and FIM efficiency (0.27±0.19 vs 0.22±0.25; *p*<0.001). In a subset analysis of admission FIM, the AFO group had a significantly higher discharge FIM compared with the No-AFO group in the low admission FIM (under 63) subgroup only. In the high admission FIM (63 or over) subgroup, the AFO group had a higher discharge FIM than the No-AFO group, but this difference was not significant. Similarly, the AFO group had a significantly higher discharge FIM than the No-AFO group after adjustment by inverse probability weighting (93.2±27.1 vs 88.0±33.7; *p*<0.001).

**Table 3:** Discharge FIM of the AFO and No-AFO groups.

	AFO	No-AFO	Cohen’s d (95% confidence interval)	p value
All patients (n = 1862)	91.0±25.9	91.3±32.3	0.02 (–0.08 to 0.12)	.85
Propensity score-matched patients (n = 792)	91.3±26.9	85.8±35.2	0.18 (0.04 to 0.32)	.02
High admission FIM patients (n = 360)	111.5±12.8	109.2±11.0	0.19 (–0.01 to 0.40)	.07
Low admission FIM patients (n = 436)	74.4±26.5	63.4±33.1	0.37 (0.17 to 0.57)	<.001
Inverse probability weighting adjusted (n = 1862)	93.2±27.1	88.0±33.7	0.17 (0.11 to 0.24)	<.001

AFO: Ankle–foot orthosis; FIM: Functional Independence Measure.

After excluding 48% (363/760) of propensity score-matched patients who lacked information about independent exercise, results showed that the AFO group performed independent exercise more than No-AFO group (69.2% [137/198] vs 55.8% [111/199]; *p*<0.001).

Post hoc power analysis of propensity score-matched pairs for the main outcome (an alpha value equal to 0.05, Cohen’s d of 0.18, and total sample size of 792 for a 2-tailed hypothesis) demonstrated an observed power of 99.6%.

## Discussion

The present study used a large rehabilitation inpatient database to investigate potential effect of AFOs on discharge FIM scores of stroke patients. Propensity score-matched analysis showed that AFO use was associated with increased FIM on discharge. Inverse probability weighting analysis showed similar results.

Many previous studies on post-stroke AFO use did not focus on functional recovery [2, 3]. Teasell et al. researched factors associated with AFO use in stroke patients undergoing rehabilitation, and showed that AFO use was associated with lower admission and discharge FIM [21]. However, the study had only a small sample size and an unadjusted univariate analysis. There is also the possibility that past studies suffered from confound effects, whereby patients for whom AFOs were indicated as necessary might have become concentrated in AFO groups [22]. The present study was unique because it conducted propensity score analysis to reduce selection bias by matching groups for some important characteristics. Propensity score-matched analysis requires a large sample size to gain statistically reliable results [23]; this was made possible by the use of the Japan Rehabilitation Database, which contains a large proportion of the stroke rehabilitation population. The post hoc power analysis showed that the sample size was sufficient to obtain a significant result if an effect is present.

Despite the fact that effect sizes (Cohen's d) were not large, this study found that AFOs might play an important role in functional recovery of stroke patients during rehabilitation. AFO groups had a higher amount of independent exercise. AFOs improve the biomechanics of standing and walking, thereby enabling the patient to move and exercise more safely, confidently, and effectively. These biomechanics may have positively affected the greater functional recovery in our study. It was possible that receiving a personal AFO promoted motivation to exercise, or that AFOs controlled the difficulty of exercise and made it easier to exercise outside the gym without therapists. It is also likely that AFOs made different types of therapy possible. Consequently, there is the possibility that prescription of an AFO increases the total discharge FIM score. However, we did not have data about the quality or quantity of independent exercise undertaken. In addition, data about independent exercise was missing for a large number of patients: therefore, care is required when determining the clinical implications of these results.

According to the subset analysis, AFO use was associated with good recovery for patients with low admission FIM. Low FIM patients seemed to have moderate to severe impairments and difficulty with exercising. Such patients had difficulty performing the exercise needed for appropriate rehabilitation. It was possible that the use of AFOs made the exercises easier for low FIM patients.

Several limitations should be acknowledged. First, the database lacks detailed information, including when AFOs were prescribed, how often the prescribed AFOs were used during rehabilitation, and whether or not patients borrowed an AFO from their gym. However, lack of these pieces of information would cause an underestimation of the effects of AFOs rather than an overestimation, and hence these are not critical limitations. Second, the study was not able to obtain detailed information on the types of AFOs used by individual patients. Although there are many types of recently designed AFOs, there are no studies on the relationship between AFO design and functional recovery in the post-acute stroke recovery stage [24], indicating further research on AFO design and functional recovery after stroke is required. Third, the Japan Rehabilitation Database only consists of voluntary samples, not random samples. We confirmed that there were no significant differences between the database and a preceding annual survey database for convalescent rehabilitation wards [11] in terms of age, admission FIM, discharge FIM, and length of stay. However, the generalizability of these findings to all stroke survivors in Japan might still be limited. Fourth, it was not a blind study and there is some doubt about the validity of the outcome assessment. However, there was no incentive for rehabilitation staff to measure the FIM score differently depending on whether AFOs had been prescribed or not. In addition, FIM is a popular measurement tool in Japanese rehabilitation hospitals, and almost all rehabilitation staff are familiar with it. Fifth, this was an observational study, and there were possibly unmeasured sources of confounding bias and residual potential biases as a result of individual therapists or hospitals. However, we used the propensity score analysis and generalized estimating equations to remove these biases as thoroughly as possible.

We conclude that our results show that prescription of AFOs was associated with improved functional recovery of stroke patients with low admission FIM following rehabilitation. Further studies are necessary to determine the optimal AFO types for stroke patient recovery.
